# Navigating the Crisis: A Cross-Sectional Survey Analysis of Resident Doctors’ Experiences of Specialty Training and Employment in Today’s NHS

**DOI:** 10.7759/cureus.102733

**Published:** 2026-01-31

**Authors:** Barbara Dorgan, Rachel Elliott, Stephen J Dorgan

**Affiliations:** 1 Rheumatology, Royal Free London NHS Foundation Trust, London, GBR; 2 Internal Medicine, Worcestershire Acute Hospitals NHS Trust, Worcester, GBR; 3 Department of Health Services Research, London School of Hygiene and Tropical Medicine, London, GBR

**Keywords:** nhs career opportunities, nhs training competition ratios, nhs workforce attrition, resident doctors’ satisfaction, uk postgraduate specialty training

## Abstract

Objective: To examine resident doctors' experiences of training conditions, career opportunities, workforce perceptions, and emotional strain in the NHS's current specialty training and local recruitment pathways.

Design: A cross-sectional, anonymous survey of UK resident doctors conducted via an online questionnaire. Quantitative and qualitative data on employment status, training opportunities, recruitment processes, and emotional well-being, from both the anonymous survey and NHS datasets, were collected and analysed via descriptive statistics and thematic analysis.

Setting: The NHS faces a deepening crisis. The demand for doctors continues to rise, yet qualified resident doctors encounter increasing barriers to entering specialty training or gaining secure employment.

Participants: A total of 288 UK resident doctors received the survey in June and July 2025. Participation was voluntary, and no incentives were offered. One hundred two surveys were completed, giving an overall response rate of 35% (N=288).

Results: Respondents reported growing competition for both specialty training and local employment posts, which is supported by NHS training and employment data. Only 39% (N=102) of respondents secured Specialty or Higher Training post, while just 8% (N=62) were successful applying for a trust-grade position despite substantial time and effort invested. Almost two-thirds (65%, N=62) reported missing out on advertised roles due to recruitment system failures. Respondents reported overwhelmingly negative emotions relating to fear, sadness, and anger, which directly impact staff morale and wellbeing. This manifests in significant stress, anxiety, and dissatisfaction with their career prospects alongside feelings of hopelessness, disillusionment, and demotivation. Over one-fifth (21%, N=102) have left the NHS or medicine altogether over the last two years.

Conclusions: Resident doctors currently face unprecedented challenges in applying to, and progressing in, NHS training and recruiting systems. These difficulties are having a profound effect on morale, well-being, and career viability, which is contributing to increased attrition from both the NHS and the medical profession. Urgent reforms to both training pathways and recruitment processes are needed to safeguard both workforce retention and high-quality patient care.

## Introduction

In recent years, the landscape for specialty medical training and employment of resident doctors in the NHS has shifted dramatically. Despite growing evidence of the urgent need to expand the medical workforce, many resident doctors are unable to progress in their careers and access training opportunities or secure local employment to meet service needs. 

The NHS remains under severe strain. Over 7.3 million patients remain on NHS waiting lists [1], and public satisfaction with the NHS is at its lowest level in over 40 years [2]. Persistent rota gaps leave 10s of thousands of shifts unfilled [3], and workforce burn-out is increasingly common [4]. While there is both a growing supply and a clear demand for more doctors, the current employment market structures appear unable of matching the supply of doctors with the demand.

A key driver of the mismatch is the shortage of training and non-training posts for resident doctors. While applications to Specialty Training have increased almost 300% since 2019, the number of training positions have increased by only 5% over the same period [5]. This imbalance has led to widespread frustration, with some groups, such as Foundation Year 1 (FY1) doctors, voting for industrial action in response to the lack of available positions to progress their careers [6].

Relatively recent policy changes have further compounded the issue. The removal of the Resident Labour Market Test (RLMT) and the expansion of UK medical school output [7] sought to address a medical workforce shortage across the NHS. However, without a dramatic and rapid increase in specialty training capacity, these two policy changes risk exacerbating the situation, worsening both competition and attrition amongst resident doctors wishing to work in the NHS and progress in their careers.

This study presents findings from a cross-sectional survey conducted in mid-2025. It examines the experiences, challenges, and perceptions of resident doctors navigating specialty training and longer-term employment pathways within the NHS.

An earlier version of this article was posted to the medRxiv preprint server on December 9th, 2025, at https://doi.org/10.64898/2025.12.07.25341779

## Materials and methods

Survey design and distribution

A 17-item online questionnaire (Table 1) was developed to collect both quantitative and qualitative data. The survey included closed questions and free-text comment sections to capture the experiences and perceptions of resident doctors at different stages of their training and employment journey. The only eligibility criteria were being (or having been) a NHS resident or foundation programme doctor within the last four years. This was validated by sending the survey link to purely clinical and work-related doctors’ WhatsApp Groups from years 2022-2025. Participants included UK and international medical graduates, both within and outside formal training programmes, working in the UK or overseas. The survey link using Google Docs was distributed via these WhatsApp groups to a total of 288 recipients between 10 June and 20 July 2025. Participation was voluntary, and no incentives were offered. 

**Table 1:** Survey tool - Online questionnaire for resident doctors The link to the online questionnaire was distributed to a total of 288 resident doctors via WhatsApp. The survey link is provided in the Appendix. ACCS – Acute Care Common Stem (run-through core training programme in acute specialties such as emergency medicine, anaesthetics, acute medicine, intensive care) CT Surgery – Core Training in Surgery (early postgraduate surgical training) CT1 – Core Training Year 1 (first year of a core training programme, usually medical or surgical) FY1 – Foundation Year 1 (first year of UK post-graduate medical training) FY2 – Foundation Year 2 (second year of UK post-graduate medical training) FY3 – Foundation Year 3 (informal term for posts taken after FY2 but before entering specialty training) GP Trainee – General Practice Trainee (doctor in specialty training for general practice) IMT – Internal Medicine Training (core medical training programme before higher specialty training) JCF – Junior Clinical Fellow (non-training hospital doctor post) LED – Locally Employed Doctor by Trust NHS – National Health Service (publicly funded healthcare system of the United Kingdom) SCF – Senior Clinical Fellow (non-training hospital doctor post, usually middle grade) SHO – Senior House Officer (traditional term for intermediate-grade resident doctors, now often used informally) SpR – Specialist Registrar (doctor in higher specialty training; now often termed ST3+) ST1 – Specialty Training Year 1 (first year of a run-through specialty training programme) ST3 – Specialty Training Year 3 (more advanced level of specialty training)

Online questionnaire	
Question 1: What best captures your current role*? *NB: At time when survey was conducted, 10 June and 20 July 2025	
Available responses Question 1:	FY1
	FY2
	In Postgraduate Specialty Training role, e.g. IMT, CT Surgery, GP Trainee, Other Specialty Trainee
	In Higher Specialty Training role (ST3 and above)
	Locally Employed Doctor, e.g. JCF, on fixed term contract (FY3 and above)
	Locally Employed Doctor, e.g. SCF, on fixed term contract (ST3 and above)
	Locum Doctor - SHO/FY3 and above (full time Locum Doctor only, not in addition to other fixed term contract or training posts)
	Locum Doctor - SpR/ST3 and above (full time Locum Doctor only, not in addition to other fixed term contract or training posts)
	Working within the NHS, but in a non-clinical or mixed role, such as Research or Teaching Fellow
	Working in private sector in the UK (as a doctor)
	Working in private sector in the UK (in a non-clinical role)
	Working abroad as a doctor
	Working abroad in a non-clinical role
	Other:
Question 2: Please select what best describes your role from September 2025 (either confirmed or most likely)	
Available responses Question 2:	FY1
	FY2
	In Postgraduate Specialty Training role, e.g. IMT, CT Surgery, GP Trainee, Other Specialty Trainee
	In Higher Specialty Training role (ST3 and above)
	Locally Employed Doctor, e.g. JCF, on fixed term contract (FY3 and above)
	Locally Employed Doctor, e.g. SCF, on fixed term contract (ST3 and above)
	Locum Doctor - SHO/FY3 and above (full time Locum Doctor only, not in addition to other fixed term contract or training posts)
	Locum Doctor - SpR/ST3 and above (full time Locum Doctor only, not in addition to other fixed term contract or training posts)
	Working within the NHS, but in a non-clinical or mixed role, such as Research or Teaching Fellow
	Working in private sector in the UK (as a doctor)
	Working in private sector in the UK (in a non-clinical role)
	Working abroad as a doctor
	Working abroad in a non-clinical role
	Other:
Question 3: Have you ever applied to any of the NHS specialty training programs outside of the Foundation Program?	
Available responses Question 3:	If YES, please indicate how many training programs in total you have applied for over the years/recruitment cycles. Answer: ___________________
	If NO, go straight to Question 10
Question 4: Have you applied to any specialty training programs for entry in 2025?	
Available responses Question 4:	If YES, tick all that apply:
	IMT1
	IMT3
	Surgery Core CT1
	Psychiatry Core CT1
	ACCS/Anaesthetics CT1
	GP Training ST1
	Paediatrics ST1
	Obs and Gynae ST1
	Other Run Through Programmes (ST1)
	Higher Specialty Training (ST3 and above)
	Other:___________________
	If NO, go straight to Question 7
Question 5: If YES to Question 4, please indicate how many applications resulted in shortlisting/ interviews for entry in 2025	
Answer: ___________________	
Question 6: If you have been shortlisted / interviewed, please indicate how many resulted in a training place offer for entry in 2025	
Answer: ___________________	
Question 7: Did you apply to any specialty training programs for entry in 2024?	
Available responses Question 7:	If YES, tick all that apply:
	IMT1
	IMT3
	Surgery Core CT1
	Psychiatry Core CT1
	ACCS/Anaesthetics CT1
	GP Training ST1
	Paediatrics ST1
	Obs and Gynae ST1
	Other Run Through Programmes (ST1)
	Higher Specialty Training (ST3 and above)
	Other:___________________
	If NO, go straight to Question 10
Question 8: If YES to Question 7, please indicate how many applications resulted in shortlisting/ interviews for entry in 2024	
Answer: ___________________	
Question 9: If you have been shortlisted / interviewed, please indicate how many resulted in a training place offer for entry in 2024	
Answer: ___________________	
Question 10: Have you applied or tried to apply for a NHS fixed term contract or permanent job, e.g. LED, JCF or SCF roles or Trust Grade Jobs over the last two years, 2023 to 2025?	
Available responses Question 10:	If YES, please answer the next questions 11 to 14
	If NO, go straight to Question 15
Question 11: If YES to Question 10, please fill in the number of jobs you were interested in (or closest estimate), but for which you could NOT submit an application, as the job application window closed early / before the advertised deadline	
Answer: ___________________	
Question 12: If YES to Question 10, please fill in the total number of applications you have submitted (or closest estimate)	
Answer: ___________________	
Question 13: If YES to Question 10, please fill in how many (or closest estimate) of these submitted applications resulted in an interview/ shortlisting	
Answer: ___________________	
Question 14: If YES to Question 10 and you have been interviewed/shortlisted for a job, please indicate how many resulted in a job offer. Otherwise leave blank.	
Answer: ___________________	
Question 15: Rate how satisfied you are with the current Postgraduate Medical Specialty Training opportunities offered in the NHS	
Rate from 1 to 10: ____________ with 1=Highly dissatisfied, 2=Very dissatisfied, 3=Dissatisfied, 4=Somewhat dissatisfied, 5=Slightly more dissatisfied but generally neutral, 6=Slightly more satisfied but generally neutral, 7=Somewhat satisfied, 8=Satisfied, 9=Very dissatisfied, 10=Highly satisfied	
Question 16: Rate how satisfied you are with the current Resident Doctor Job opportunities (outside of training programs) offered in the NHS	
Rate from 1 to 10: ____________ with 1=Highly dissatisfied, 2=Very dissatisfied, 3=Dissatisfied, 4=Somewhat dissatisfied, 5=Slightly more dissatisfied but generally neutral, 6=Slightly more satisfied but generally neutral, 7=Somewhat satisfied, 8=Satisfied, 9=Very dissatisfied, 10=Highly satisfied	
Question 17: Please describe in a few words how the current Postgraduate Medical Specialty Training and wider resident doctor job situation in the NHS makes you feel	
Answer: _________________________________________________________________	
Please provide some further info about your Medical Training and select as applicable (optional):	
Available responses:	I am a UK Medical School Graduate (UK national)
	I am a UK Medical School Graduate (non-UK national)
	I am an International Medical Graduate (UK national)
	I am an International Medical Graduate (non-UK national)
	Other: ___________________
What year did you graduate from Medical School?	
Answer: ____________________	
When did you or will you complete your Foundation Training / FY2?	
Year/Month: _________________	

Data collection and analysis

Quantitative survey responses were analysed descriptively. Free-text comments were examined using an inductive thematic analysis approach to identify recurring patterns, sentiments, and emerging themes. Two researchers independently reviewed responses to enhance consistency, and any discrepancies were resolved through consensus and discussion.

To put the survey data in context, we performed a secondary analysis of publicly available NHS workforce and training data for the period 2019-2025 [8,9]. The published data is in a very detailed format and too granular; it required extensive data aggregation and analysis by the authors before it could be used.

Data availability

The data generated during the research and analysis are not available publicly but are available from the corresponding author on a reasonable request.

Strengths and limitations of this study

This study provides timely evidence on resident doctors’ experiences of current NHS training and employment pathways during a period of unprecedented competition. A mixed-methods approach, combining survey data with qualitative analysis and publicly available NHS workforce datasets, strengthens the depth and contextual relevance of the findings.

Selection bias is possible, as doctors with negative experiences may have been more likely to participate. This is compounded by convenience sampling via WhatsApp groups overrepresenting early-career doctors (with a high proportion of UK graduates). A resulting sampling bias limits generalisability to all 79,000 resident doctors. There is a reliance on self-reporting for some of the data on non-training employment opportunities with possible recall-bias. This had to be accepted as unavoidable as further validation by external sources was outside of the scope of this work. The design of the study limits its inference of causality, which is neither proven nor modelled, as this would be beyond the scope of this study. Any causality inferred from the context is intended by the authors to be suggestive.

Ethics

All data were collected anonymously. No personally identifiable information was recorded. The research involved health care staff, recruited by virtue of their professional role. Respondents were fully informed in advance about the purpose of the study and provided implied consent by voluntarily completing the survey. The study did not involve patients or access to identifiable health records. 

Ethical approval was not required under NHS Health Research Authority guidance.

Guidelines

STROBE reporting guidelines [10] were used when drafting and revising this manuscript and the STROBE reporting checklist [11] was completed prior to submission. 

## Results

Respondent demographics

A total of 102 surveys were completed, giving an overall response rate of 35% (N=288). Table 2 summarises survey participants’ demographics and roles at the time of the survey (June/July 2025) and future roles from September 2025, with additional detail about their level of experience provided in Table 3. Most respondents (80%, N=102) were resident doctors who graduated medical school between 2021 and 2023 and completed their Foundation Training Programme between 2023 and 2025. The majority (89%, N=102) were graduates of UK medical schools. Overall, 39% (N=102) expected to hold a formal Specialty Training post in Autumn 2025, with 33% (N=102) in Postgraduate Specialty Training, an increase from 22% (N=102) the previous year, and 6% (N=102) of respondents in Higher Specialty Training, up from 4% (N=102) in the previous year.

**Table 2:** Respondent characteristics – Country of qualification and role Analysis of online questionnaire completed by resident doctors in June / July 2025 for this survey *N = 102

	June-July 2025		After Sept. 2025	
	n	Percentage*	n	Percentage*
UK medical school graduates:				
UK nationals	83	81%	83	81%
Non-UK nationals	8	8%	8	8%
Non-UK medical school graduates:				
UK national	2	2%	2	2%
Non-UK national	7	7%	7	7%
No information given:	2	2%	2	2%
Role:				
Foundation Year 1	3	3%	0	0%
Foundation Year 2	18	18%	3	3%
Postgraduate speciality training	22	22%	34	33%
Higher speciality training	4	4%	6	6%
Locally employed doctor	29	28%	21	21%
Locum doctor	14	14%	17	17%
Doctor working abroad	11	11%	13	13%
Non-clinical role	1	1%	2	2%
Full-time student	0	0%	2	2%
Other:	0	0%	4	4%

**Table 3:** Respondent characteristics – Level of experience Analysis of online questionnaire completed by resident doctors in June / July 2025 for this survey *N = 102

Year	Graduated Medical School		Completed Foundation Training	
	n	Percentage*	n	Percentage*
2017 and earlier	6	6%	6	6%
2018	2	2%	1	1%
2019	5	5%	1	1%
2020	5	5%	0	0%
2021	11	11%	5	5%
2022	54	53%	4	4%
2023	16	16%	12	12%
2024	3	3%	51	50%
2025	0	0%	18	18%
2026	0	0%	4	4%

More than half of respondents (58%, N=102) remained outside formal training pathways. 21% (N=102) reported working as Locally Employed Doctors, down from 28% (N=102) the previous year, while 17% (N=102) were working as locum doctors, up from 14% (N=102). Over one in five respondents (21%, N=102) reported leaving the NHS altogether, an increase from 12% (N=102) the previous year. Of the 21% (N=102) leaving the NHS, 13% (N=102) were leaving the UK, while 8% (N=102) indicated they would no longer work as doctors after September 2025.

Specialty training opportunities

Table 4 shows the overall share of respondents applying for Specialty Training increased from 40% (N=102) in 2024 to 51% (N=102) in 2025. However, success rates in securing a training role or an interview declined. In 2025, 63% (N=79) of applications failed to result in a training position compared with 57% (N=60) in 2024, and less than half (48%, N=79) of applications resulted in an interview in 2025, down from 63% (N=60) the previous year.

**Table 4:** Speciality training applications, success and failure rates – by year *N=102 in both 2024 and 2025; **N=60 in 2024 and N=79 in 2025

Speciality training	Recruitment Cycle Year			
	2024		2025	
	n	%	n	%
Total individuals applying for a speciality training role*	41	40%	52	51%
Total speciality training applications made in-year	60	n/a	79	n/a
Applications made that resulted in:				
An interview**	38	63%	38	48%
A training post offer**	26	43%	29	37%
No offer of a training post**	34	57%	50	63%

**Figure 1:**
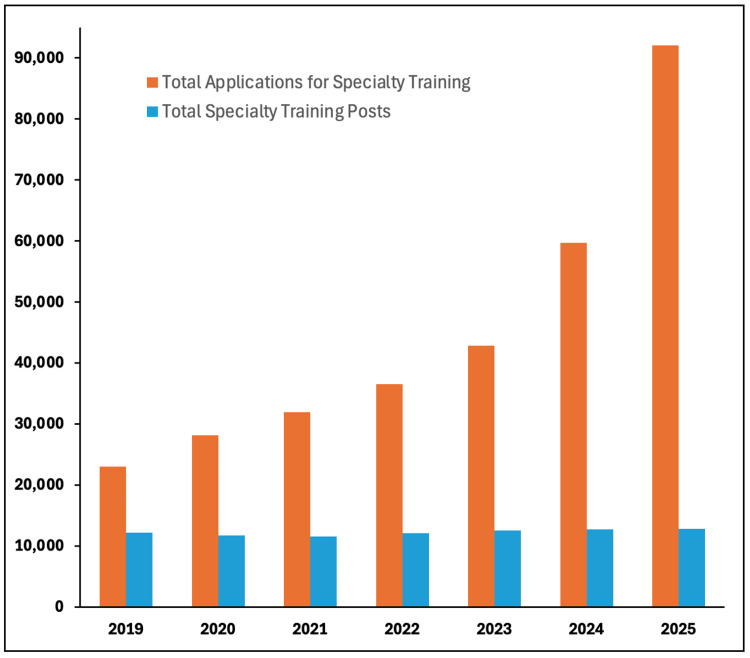
NHS England: Speciality training applications and available posts, 2019-2025 Data aggregation and analysis by authors. Data source: Health Education England. Competition Ratios 2021-2025 [8]

Figure 1 illustrates the widening gap between the number of Speciality Training applications and the number of available posts. Between 2019 and 2025, the number of applications increased by 299% (from 23,040 to 91,999), while the number of training posts rose by only 5% from 12,175 to 12,833 [8].

**Figure 2:**
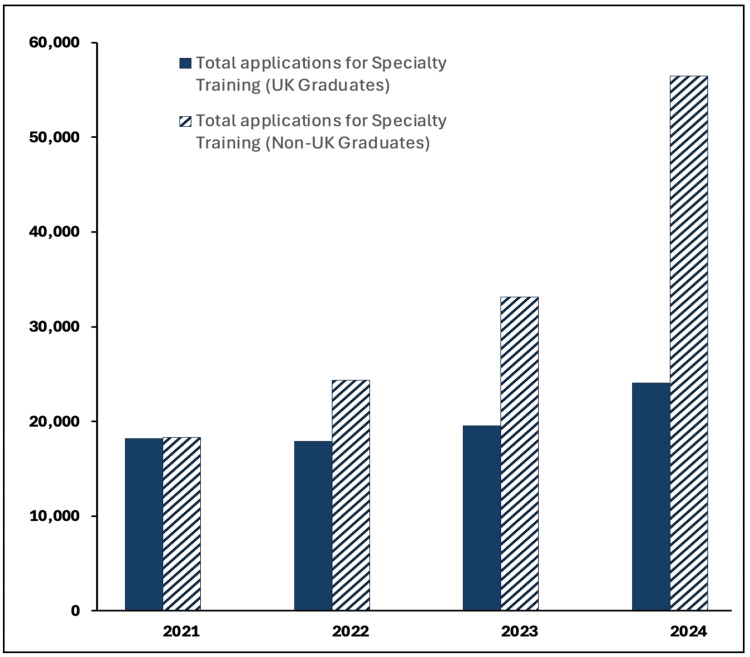
Specialty training applications by country of qualification, 2021-2024 Data aggregation and analysis by authors. Data source: Health Education England. Equality and diversity recruitment data 2021-2024 [9]

Figure 2 shows that the recent rise in Specialty Training applications is largely driven by non-UK graduates. Applications from non-UK graduates increased by 208%, from 18,335 in 2021 to 56,453 in 2024, while UK-graduate applications have seen a much more moderate increase of 32%, from 18,195 to 24,068 over the same period [9].

Non-training employment opportunities

**Figure 3:**
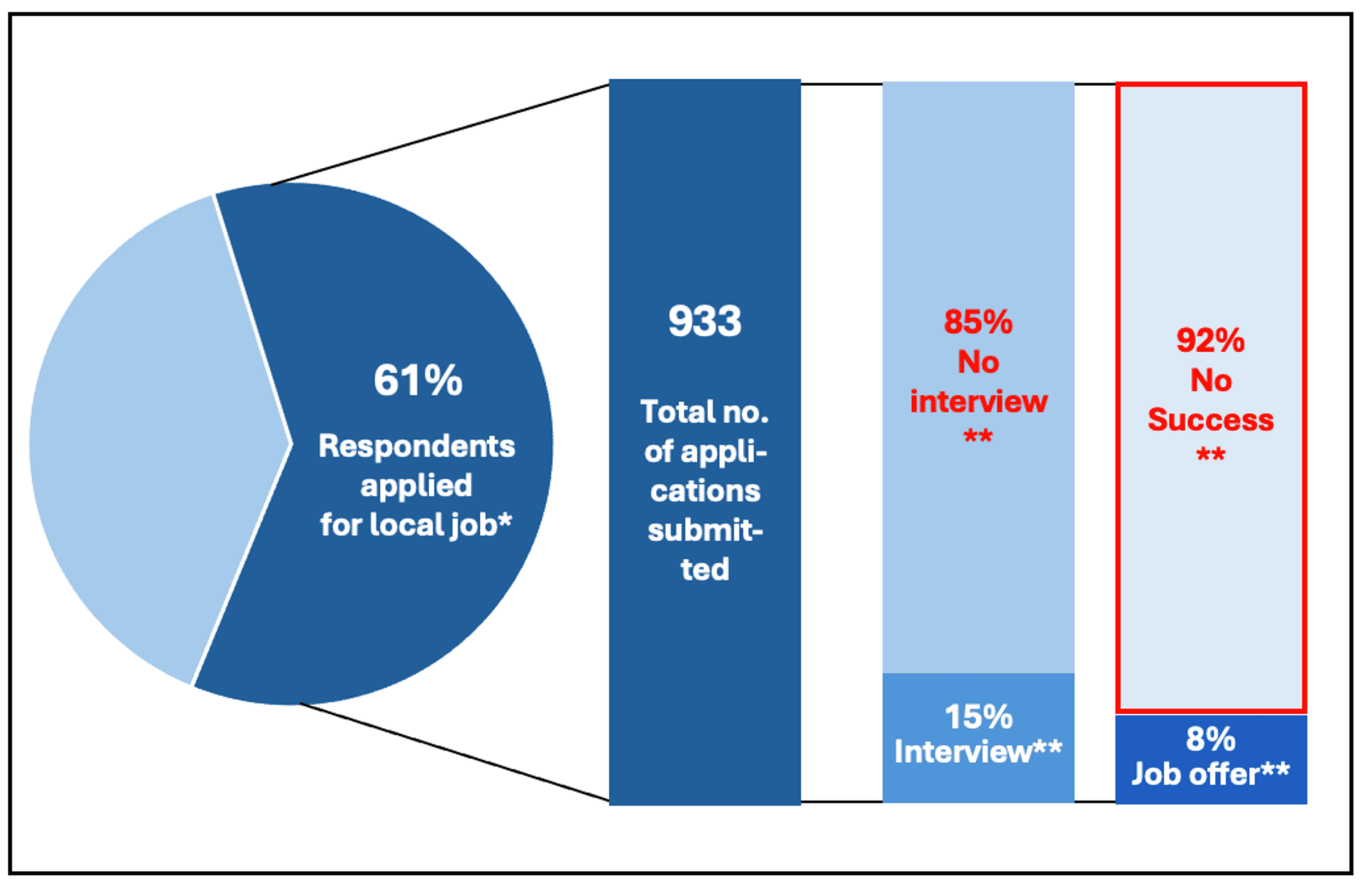
Local trust-based job application success/failure rates, 2023-2025 Analysis of online questionnaire completed by resident doctors in June / July 2025 for this survey * Applications submitted over the last two years, 2023-2025; N=62 ** N=933

The secondary impact of limited specialty training post availability upon the wider job market appears significant. Over the past two years, 61% (N=102) of respondents applied for local trust-based roles, submitting a total of 933 applications between them. Only 15% (N=933) of those applications were put forward for an interview, and just 8% (N=933) of those applications resulted in a job offer. Overall, 92% (N=933) of applications submitted for a local post were unsuccessful and did not result in any position (Figure 3).

**Figure 4:**
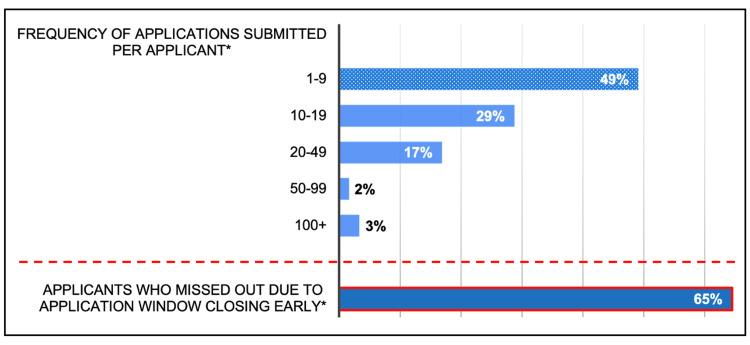
Frequency of applications for local trust-based roles, 2023-2025 Analysis of online questionnaire completed by resident doctors in June / July 2025 for this survey *Applications submitted over the last two years, 2023-2025; N=62

Competition for these local trust-based roles was intense. Over half of those applying for trust-based roles (51%, N=62) submitted 10 or more applications between 2023 to 2025, with 3% (N=62) of respondents submitting over 100 applications. In addition, almost two-thirds (65%, N=62) of those applying for trust-based roles reported missing out on one or more advertised posts because the online application window closed prematurely, i.e., ahead of the advertised deadline, preventing them from applying; the total number of missed opportunities is reported as 386 times by the respondents (Figure 4).

Satisfaction levels with training and job opportunities

**Figure 5:**
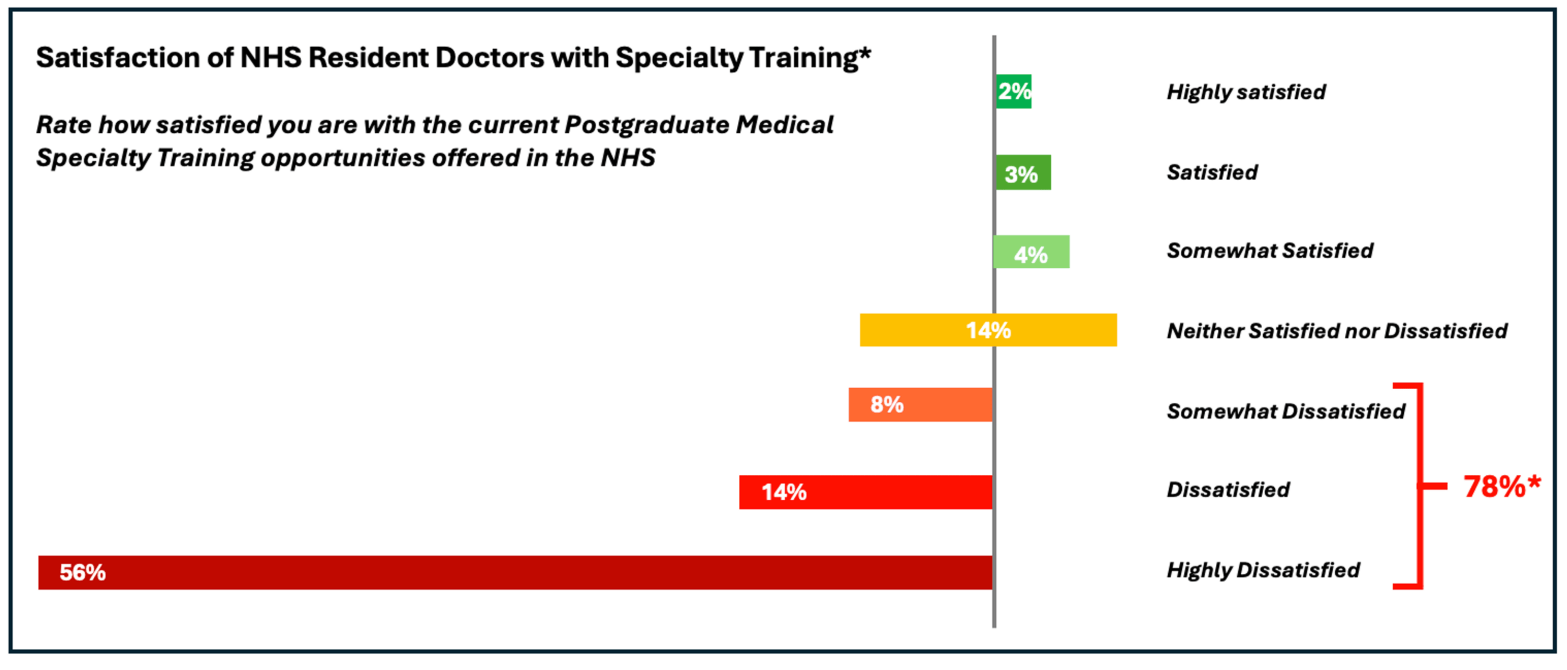
Satisfaction of NHS resident doctors – Specialty Training, 2025 Analysis of online questionnaire completed by resident doctors in June / July 2025 for this survey *N=102 Method: Semantic Mapping for visualisation in Likert-Scale bars. Original 10-point scale with 1=Completely dissatisfied, 2=Very dissatisfied, 3=Dissatisfied, 4=Somewhat dissatisfied, 5=Slightly more dissatisfied but generally neutral, 6=Slightly more satisfied but generally neutral, 7=Somewhat satisfied, 8=Satisfied, 9=Very dissatisfied, 10=Completely satisfied was mapped to 7-Point Likert Label based on intended meaning rather than exact spacing using the following conversion: 1-2 to Highly Dissatisfied (1), 3 to Dissatisfied, 4 to Somewhat Dissatisfied (3), 5-6 to Neutral (4), 7 to Somewhat Satisfied (5), 8 to Satisfied (6), 9-10 to Highly Satisfied (7)

In total, 78% (N=102) of resident doctors were unhappy (highly dissatisfied, dissatisfied, somewhat dissatisfied) with current Specialty Training opportunities (Figure 5). 

**Figure 6:**
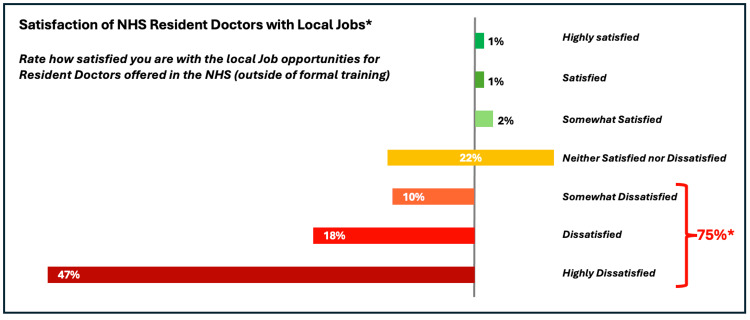
Satisfaction of NHS resident doctors – Local jobs, 2025 Analysis of online questionnaire completed by resident doctors in June / July 2025 for this survey *N=102 Method: Semantic Mapping for visualisation in Likert-Scale bars. Original 10-point scale with 1=Completely dissatisfied, 2=Very dissatisfied, 3=Dissatisfied, 4=Somewhat dissatisfied, 5=Slightly more dissatisfied but generally neutral, 6=Slightly more satisfied but generally neutral, 7=Somewhat satisfied, 8=Satisfied, 9=Very dissatisfied, 10=Completely satisfied was mapped to 7-Point Likert Label based on intended meaning rather than exact spacing using the following conversion: 1-2 to Highly Dissatisfied (1), 3 to Dissatisfied, 4 to Somewhat Dissatisfied (3), 5-6 to Neutral (4), 7 to Somewhat Satisfied (5), 8 to Satisfied (6), 9-10 to Highly Satisfied (7)

There was similar dissatisfaction with employment outside formal training, with 75% (N=102) of respondents unhappy with available NHS job opportunities (Figure 6). 

Qualitative sentiment analysis

To complement the quantitative data, participants were asked: *“Please describe in a few words how the current Postgraduate Medical Specialty Training and wider Resident Doctor job situation in the NHS makes you feel?”* 

Eighty-eight respondents (86%, N=102) provided free-text comments, which were analysed thematically. The thematic insights are structured around identified key themes, reports, and selected illustrative quotes from respondents and summarised in Table 5.

**Table 5:** Thematic Insights – Key themes, reports and representative quotes Analysis of online questionnaire completed by resident doctors in June / July 2025 for this survey Free-text comments were examined using an inductive thematic analysis approach to identify recurring patterns, sentiments, and emerging themes

Key Theme	What doctors report	Representative Quotes
Emotional strain and mental health	Feeling broken, emotionally exhausted and on the edge of giving up medicine; suffering deteriorating mental health.	“I feel mentally and emotionally exhausted from trying to progress in the NHS.”
		“Frustrated and powerless, undervalued and left out cold, soul and confidence destroying. Had to stop applying as too detrimental for my mental health.”
		“Hopeless, anxious, looking into other careers.”
		“Scared and worried about future, helpless, trapped.”
		“Repeated rejections have made me feel broken, anxious, and on the edge of giving up medicine.”
		“It’s an absolute scandal. My mental health has suffered massively, my confidence as a doctor is shattered to pieces, I won’t be able to continue in medicine.”
Perception of being undervalued and not worthy of investment in future	Feeling undervalued and replaceable, seeing little investment in their future, and viewing medicine as a questionable career choice.	“Undervalued. doctors are treated as numbers, not people with lives.”
		“I feel invisible, undervalued, and replaceable.”
		“Demoralised, unwanted, frustrated. Working so hard in a broken system and having to face the fact, the same system can’t facilitate your ongoing training.”
		“It is difficult not to feel disheartened and like I have made a mistake in choosing this career.”
		“To be exiting F2 without a formal job despite so many applications is so discouraging and just makes me feel so ready to leave medicine now.”
		“Tricked, lied to, used and discarded.”
Job insecurity and extreme competition	Impossible to secure training posts, unrealistic competition ratios and lack of career progression.	“It feels nearly impossible to get a training post; the process is overly competitive and demoralising.”
		“It’s extremely disheartening to work so hard and not feel appreciated and be anxious about getting to the next stage of training.”
		“Undervalued, exploited, hopeless. Increased competition ratios every year with no attempt to fix it.”
		“It makes me feel that there is little hope for entering speciality training and very frustrated that I have invested so much time, energy and money into a career that is supposed to offer job security & career progression but has done quite the opposite.”
		“Uncertain, uneasy without any job security. So-called lifeline learning without lifelong work!”
		“Frustrated, upset and feel stuck at my current level as I’m unable to progress in training.”
Broken by process and “survivors’ guilt”	Even if successful, doctors feel demotivated and broken by the toll it took, the need to settle for less desirable opportunities and the guilt in seeing other colleagues’ struggles.	“I am in training but I despair for those locked out and facing unemployment.”
		“I have taken a CT1 surgical job that will require more than an hour commute each way. I am doing two specialities that I have no interest in pursuing further and will leave me struggling to find an ST3 post I want to train in.”
		“It feels bleak that despite having two Cambridge degrees and 3 years experience as a doctor working more than full time hours I am grateful to be in a job that I would have no interest in applying for outside of a national training programme.”
		“It was so difficult to get into ST1, and I felt so disrespected throughout the whole process I ended up turning down my offer and decided to stay in New Zealand where you feel wanted and valued and supported in your career.”
Systemic issues and mismanagement	A lack of workforce planning, a mismatch of roles and jobs meeting the demand from doctors, prioritisation of service provision over education.	“Complex, multi-faceted but compounded by short-sighted workforce planning, coalescing in a minefield for all.”
		“Hopeless. There are rota gaps in every speciality and hospital and there are competent residents willing to do those jobs, yet the recruitment system means that those gaps go unfilled.”
		“Too much service provision in training posts and no training.”
		“Demoralised, it’s sad to see people unemployed despite having understaffed working conditions.”
		“Rotational training is awful. Training is more about service provision. Rotas are heavy with out of hours work. Departments not taking pride in trainees.”
		“Training is at times fragmented and often training and teaching opportunities are missed due to poor staffing and pressures on the NHS.”
Overwhelmed recruitment systems	Overburdened, failing recruitment systems, not fit for purpose and seen as unfair, untransparent and not selecting for the best candidates.	“I have been unable to even hear back from fellow jobs when applying, yet my CV / level is perfect for some of these jobs.”
		“Bots are causing a lot of issues. Jobs are advertised, hundreds of applications are received, but very few if any are from people suited to the post advertised.”
		“Definition of madness. Does not choose the best/most suitable doctors, you have to pay to play and rewards pen pushers.”
		“Many changes especially in IMT recruitment over the last 6 years have meant that many resident doctors are now struggling to get into training and even risk of being unemployed, which is extremely disheartening and disappointing.”
Missed opportunity and inequity	Displacement of local graduates, recruitment not giving sufficient weight to relevant clinical experience, expensive upskilling requirements.	“Bottlenecks, underfunding and political short-sightedness are threatening the future of healthcare.”
		“International recruitment has unintentionally displaced competent local graduates.”
		“Frustrated at the NHS’ desperation for doctors and yet the lack of training opportunities for UK graduates. This makes no sense to me at all.”
		“After all this work and perceived rota gaps, nothing is being done to help the right people get in the right jobs.“
		“It is very costly to get to the next step through all the courses etc required to demonstrate ‘commitment to specialty’.”
		“Too much extra-curricular non-clinical CV work required to achieve a core training place!”
Exodus from the NHS and medicine	Intent to leave the NHS or the UK, migrating to countries with better conditions. Conflicted about choices and feeling forced into them.	“Many of us are looking abroad or outside of medicine for job security, appreciation, respect, and to preserve our overall mental wellbeing.”
		“Desperate. I feel as if I have no choice but to leave the UK, despite wanting to stay.”
		“Like leaving medicine. Grad med was a total waste of time resulting in financial insecurity and stress without any job satisfaction.”
		“I am considering non-medical career paths but am finding it hard to fully commit to this, having trained so long to become a doctor. I also feel guilty for leaving. On the one hand, I want to help patients and fiercely believe in the NHS’ core principles, but on the other, I need to protect myself as I cannot work in the NHS without it breaking me.”

The qualitative analysis identified a high prevalence of negative emotions with clear implications for morale and well-being. Three dominant emotional categories emerged: 1) ‘fear’, reflected in reports of anxiety, worry, insecurity, and uncertainty; 2) ‘sadness’, encompassing feelings of hopelessness, despair, and being undervalued; and 3) ‘anger’ often intertwined with frustration.

## Discussion

Our findings demonstrate a clear employment crisis for resident doctors in the NHS, which has been developing over the past six years. Three interrelated factors underpin the current situation: (1) the number of Specialty Training posts has remained largely static; (2) applications for training posts have risen sharply; and (3) recruitment processes are overwhelmed and inadequate. 

Over half of survey respondents are currently outside formal training roles due to increased competition for those roles. In the 2025 recruitment cycle, 63% (N=79) of applicants failed to secure any training post, compared with 53% (N=60) in 2024, and fewer than half (48%, N=79) were invited to interview in 2025, down from 63% (N=60) previously. These findings indicate that the selection process is under significant strain, a trend likely to continue unless substantial reforms are implemented.

The lack of training posts is not a new phenomenon. Between 2013 and 2020, there were approximately 2.5 applicants per specialty training post annually [8]. The removal of the Resident Labour Market Test in 2020 contributed to a sharp rise in competition, increasing applicants per post from 1.9 in 2019 to 7.2 in 2025, largely driven by a tripling of applications from non-UK graduates. Non-UK graduates now account for 70% of all applications [9], overwhelming a recruitment system that is unable to process the now 92,000 applications for fewer than 13,000 available posts [5,8].

Poorly conceived solutions to manage the explosion in training post applications have made the situation even worse. For example, in Internal Medicine, available training posts have remained static at approximately 1,600 annually since 2015, while interview capacity is limited to around 4,000 [12]. To manage the huge number of applications, interview candidates are selected based upon self-scoring exercises with minimal verification. In fact, applicants are instructed to not provide supporting evidence, unless specifically requested, which they are assured is “highly unlikely” [13]. Other Specialty Trainings such as GP Training or Psychiatry have eliminated interviews entirely. These measures, while intended to manage workloads, risk diluting selection rigour and potentially compromising patient care.

The lack of training posts and the growing number of doctors unable to secure Speciality Training roles is increasing competition for non-training roles. More than half of respondents applying for local posts submitted over 10 applications, and some submitted over 100, yet success rates remain low. Fewer than one in 12 applicants secured a local role, with inadequate recruitment processes being reported as one of the key drivers of this challenging situation. 

Automated systems now prioritise application speed over experience. Two-thirds of all respondents (65%, N=62) applying for such local jobs reported missing out on roles because the application window closed earlier than advertised, and before they could apply. Increasingly, automated ‘bots’ are immediately applying for roles online as soon as they are advertised despite the applicants being wholly unqualified. In one example from the survey, applications for a role closed automatically after just one and a half hours rather than the advertised three weeks, despite 90% (N=500) of the applications received being substandard. These automated systems effectively exclude highly qualified candidates and undermine any argument that a more competitive labour market will improve candidate quality [14].

These system inefficiencies drive a substantial loss of public investment. The £230,000 taxpayers spend to educate and train each doctor [15] is lost when skilled resident doctors leave the NHS due to poor workforce planning and failing training and employment pathways. Our findings of high attrition rates of 21% (N=102) aligns with more current studies, such as the Royal College of Physicians’ 2025 Study reporting 35% (N= >500 and <1202) of doctors do not plan on working in the NHS in five years’ time [16].

Our survey highlights the broader impact on resident doctors’ morale and wellbeing. Over three-quarters of respondents reported dissatisfaction with both Specialty Training and wider job opportunities, even though 39% (N=102) of the cohort held a Specialty Training post in September 2025. This suggests widespread frustration with the effort required and complexity of recruitment processes. Free-text responses reveal pervasive negative emotions of anxiety, stress, hopelessness, and frustration. These emotions indicate significant psychological distress amongst respondents and appear to contribute to burnout and demoralisation within the workplace. Doctors report struggling with managing both time-consuming application processes and excessive demands by recruiters to demonstrate non-clinical achievements, possibly undermining their ability to recuperate and provide the best patient care. 

Together, these observed associations highlight significant challenges and emotional burden experienced by resident doctors navigating current NHS training and employment pathways.

While policy makers have acknowledged the crisis, and the Chief Medical Officer for England has recommended an overhaul of medical training [17], implementation is unlikely to occur quickly enough to support the current cohort of resident doctors. Without urgent reform, workforce attrition, low morale, and ultimately compromised patient care are likely to continue.

## Conclusions

This study identifies a growing workforce challenge within the NHS, particularly among resident doctors. Competition for Specialty Training posts remains intense, with many applicants reapplying in later cycles, placing further strain on recruitment systems. Current processes struggle to manage the volume of applications while maintaining fairness and transparency, contributing to declining morale and well-being among doctors. These systemic pressures now threaten the stability of the medical workforce and the effectiveness of patient care. Urgent and coordinated reform is essential to expand training capacity, strengthen recruitment systems, and ensure the NHS retains and fully utilises its skilled doctors for the future.

## Appendices

The survey can be found at https://docs.google.com/forms/d/1NZd2Kx0SbRMmX1E3-TjD0UXa6kqLPZ9pEKhA29n_kVw/viewform?pli=1&pli=1&pli=1&edit_requested=true

## References

[1] (2025). NHS waiting list hits two-year low as staff work to 'turn the tide'. https://www.england.nhs.uk/2025/06/nhs-waiting-list-hits-two-year-low-as-staff-work-to-turn-the-tide/.

[2] Taylor B, Lobont C, Dayan M, Merry L, Jefferies D, Wellings D (2025). Public satisfaction with the NHS and social care in 2024 (BSA): results from the British Social Attitudes survey. https://www.kingsfund.org.uk/insight-and-analysis/reports/public-satisfaction-nhs-social-care-in-2024-bsa.

[3] (2025). BMA investigation finds more than 32,000 doctors' shifts unfilled in hospitals in London in six months. https://www.bma.org.uk/bma-media-centre/boris-johnson-s-covid-inquiry-evidence-was-a-masterclass-in-double-speak-says-bma-1.

[4] Buckingham N (2025). NHS workforce in a nutshell. https://www.kingsfund.org.uk/insight-and-analysis/data-and-charts/nhs-workforce-nutshell.

[5] Bowie K (2025). Competition ratios: hundreds of doctors compete for some specialty training posts, show "scandalous" NHS data. BMJ.

[6] O'Dowd A (2025). First year resident doctors in England vote to strike over job shortages. BMJ.

[7] Ferreira T (2024). Escalating competition in NHS: implications for healthcare quality and workforce sustainability. Postgrad Med J.

[8] (2025). Competition ratios. https://medical.hee.nhs.uk/medical-training-recruitment/medical-specialty-training/competition-ratios.

[9] (2025). Equality and diversity recruitment data: country of qualification, applications made. https://medical.hee.nhs.uk/medical-training-recruitment/medical-specialty-training/equality-and-diversity.

[10] von Elm E, Altman DG, Egger M, Pocock SJ, Gøtzsche PC, Vandenbroucke JP (2007). Strengthening the Reporting of Observational Studies in Epidemiology (STROBE) statement: guidelines for reporting observational studies. BMJ.

[11] (2025). STROBE checklist for cross-sectional studies. https://www.strobe-statement.org/checklists/.

[12] (2025). JRCPTB Position Statement on Recruitment Plans for IMT in 2026. https://www.thefederation.uk/sites/default/files/uploads/JRCPTB%20position%20statement%20on%20recruitment%20plans%20for%20IMT%20in%202026.pdf.

[13] (2025). IMT recruitment: application scoring. Reviewed 30 August.

[14] Burch D (2025). We need more unemployed doctors. The Spectator.

[15] (2025). More undergraduate medical education places: expanding medical education to train up to 1,500 extra doctors each year in England. http://www.gov.uk/government/news/more-undergraduate-medical-education-places.

[16] Douthwaite H, Shamsher Ahmed M, Ariyo M, Arun B, Parker H (2025). Joint Royal Colleges of Physicians’ Position Statement: The Voice of Our Next Generation: The Results of Our 2025 National Survey of Resident Doctors. https://www.rcp.ac.uk/policy-and-campaigns/policy-documents/the-voice-of-our-next-generation-the-results-of-our-2025-national-survey-of-resident-doctors/#:~:text=20%25%20were%20unsure.-,Burnout%2C%20pay%20and%20workload%20are%20driving%20doctors%20to%20consider%20leaving,%2C%20with%20another%2034%25%20undecided.

[17] (2025). Medical training review: phase 1 diagnostic report. http://www.england.nhs.uk/publication/the-medical-training-review-phase-1-diagnostic-report.

